# The promise of zero-shot learning for alcohol image detection: comparison with a task-specific deep learning algorithm

**DOI:** 10.1038/s41598-023-39169-4

**Published:** 2023-07-23

**Authors:** Abraham Albert Bonela, Aiden Nibali, Zhen He, Benjamin Riordan, Dan Anderson-Luxford, Emmanuel Kuntsche

**Affiliations:** 1 Centre for Alcohol Policy Research, La Trobe University, Melbourne, Australia; 2 Computer Science and Information Technology, La Trobe University, Melbourne, Australia

**Keywords:** Public health, Epidemiology

## Abstract

Exposure to alcohol content in media increases alcohol consumption and related harm. With exponential growth of media content, it is important to use algorithms to automatically detect and quantify alcohol exposure. Foundation models such as Contrastive Language-Image Pretraining (CLIP) can detect alcohol exposure through Zero-Shot Learning (ZSL) without any additional training. In this paper, we evaluated the ZSL performance of CLIP against a supervised algorithm called Alcoholic Beverage Identification Deep Learning Algorithm Version-2 (ABIDLA2), which is specifically trained to recognise alcoholic beverages in images, across three tasks. We found ZSL achieved similar performance compared to ABIDLA2 in two out of three tasks. However, ABIDLA2 outperformed ZSL in a fine-grained classification task in which determining subtle differences among alcoholic beverages (including containers) are essential. We also found that phrase engineering is essential for improving the performance of ZSL. To conclude, like ABIDLA2, ZSL with little phrase engineering can achieve promising performance in identifying alcohol exposure in images. This makes it easier for researchers, with little or no programming background, to implement ZSL effectively to obtain insightful analytics from digital media. Such analytics can assist researchers and policy makers to propose regulations that can prevent alcohol exposure and eventually prevent alcohol consumption.

## Introduction

Alcohol use is an ongoing health concern and represents 5.1% of the global burden of disease^1^, causing a significant negative impact on individuals and the economy^2^. Despite the harms and the call from the World Health Organisation to reduce alcohol exposure^1^, alcohol imagery is practically unavoidable in both the physical^3^ and the digital world. For example, alcohol exposure is common on social media sites (~ 2% of posts^4^), in films (25 times per popular movie^5^), in online advertising^1^, and in popular music^6^.

This is concerning because of the reported link between the amount of alcohol we are exposed to and alcohol use^7,8^. For example, children who are more exposed to alcohol in films are more likely to initiate alcohol use earlier^9^. Additionally, it appears that there is a link between exposure to alcohol-related social media posts and alcohol use^7^. A recent study measured how much alcohol young adults were exposed to on their Instagram feeds during the transition to university and found that those who were exposed to more alcohol content were more likely to report drinking more over time^10^.

Given the risks associated with alcohol exposure in digital media, it is important that accurate estimates of alcohol exposure are available. Although there is a growing body of literature which has investigated alcohol exposure in media, to date, few studies have been conducted at a sufficient scale to provide accurate estimates of prevalence or trends in alcohol exposure over time. This is because traditionally studies have relied on manual annotation of digital media^10^. For example, in their project measuring the link between exposure to alcohol-related posts on Instagram and subsequent alcohol use, LaBrie et al.^10^ collected and hand-annotated 89,917 images to estimate how much alcohol people were exposed to on Instagram. Additionally, as more alcohol imagery exists than ever before and we are spending more time online, we need more sophisticated ways to analyse and provide evidence of the amount of alcohol exposure in digital media. To our knowledge, Alcoholic Beverage Identification Deep Learning Algorithm (ABIDLA)^11^ was the first deep learning algorithm that was able to identify beer cup, beer bottle, beer can, wine, champagne, and “other” from digital images with 73.8% accuracy and achieved 85.2% accuracy for the binary classification (i.e., whether an image contains any type of alcoholic beverage). More recently, researchers developed ABIDLA2^12^ which extended ABIDLA’s capabilities^11^ by including a more comprehensive list of beverage categories: cider, brandy, whiskey, cognac, and cocktails. ABIDLA2 achieved an accuracy of 77.0% for identifying eight beverage categories and 87.7% accuracy when distinguishing between alcohol images versus non-alcohol images. Although supervised deep learning models like ABIDLA and ABIDLA2 can automatically identify alcoholic beverages from images, they are burdensome to train as they require lot of manually annotated data. For example, ABIDLA2 was trained using more than 160,000 manually annotated images. Furthermore, for the identification of new categories (e.g., new beverages or other substances) additional manual annotation and further model training is required. This is one of the major drawbacks of traditional supervised deep learning algorithms.

An alternative to traditional supervised learning is zero-shot learning (ZSL). In ZSL, a model is first trained on a general task using data that is easily obtainable at a large scale (e.g., matching images to their corresponding caption on the web). Once the model has developed an understanding of images, natural language phrases, and the relationship between them, it can then be deployed to perform a specific target labelling task (e.g., identifying whether an image contains beer cups or wine, etc.) without the need to annotate any images in the target task. ZSL effectively allows users to ask all kinds of questions about an image through phrases (or captions) as target labels, without the need to do any further model training, such as whether the image contains happy or sad people drinking. Users have been trying to perform ZSL for many years but until recently the accuracy of ZSL was much lower than deep learning models trained via traditional supervised learning with task-specific ground truth annotations^13^.

In the context of images and text, the deep learning model called Contrastive Language-Image Pretraining (CLIP)^13^ was trained on 400 million image and caption pairs collected from a variety of publicly available sources on the internet. Without the requirement of any further training using labelled data and just by performing ZSL using phrases as class labels, CLIP outperformed a model trained using supervised learning (supervised training of a linear classifier on top of ResNet-50 features) for 16 out of 27 public image classification datasets^13^. This is a revolutionary finding because performing ZSL does not require any further labelled data for training and can produce performance compared to state-of-the-art supervised learning models by just using specific phrases as class labels since it relies on the knowledge acquired by the foundation model (CLIP) trained on a large dataset. In addition, implementing ZSL requires fewer computational resources and expertise and may be an accessible alternative to traditional methods (e.g., manual annotations) for researchers interested in quantifying alcohol exposure.

Despite the promise of ZSL, it remains unclear whether ZSL can outperform a deep learning algorithm trained specifically for the task of alcohol beverage identification. Therefore, in this study we aim to investigate the performance of a ZSL model compared to a deep learning algorithm specifically trained to identify alcoholic beverages in images (ABIDLA2)^12^. In order to implement ZSL using CLIP, a researcher must provide phrases (or captions) as target labels for ZSL. Given that the ZSL performance of CLIP is sensitive to phrases and may require phrase engineering^13^. Thus, in this study we also aim to investigate two sets of phrases, *name-based phrases* (e.g., “beer bottle” which is just a contextless class name) and *descriptive-phrases* (e.g., “photo of a person drinking a bottle of beer”), to determine the performance differences between the two approaches. We hypothesise that *descriptive-phrases* outperform *name-based phrases* because the former is similar to the image captions originally used to train CLIP.

## Methods

### Background on zero-shot learning versus supervised learning

In essence, ZSL can perform image classification just like a supervised learning approach can. However, one fundamental difference between supervised learning and ZSL is that a model trained using supervised learning can only classify images belonging to a fixed set of classes. This is because, fundamentally, a traditional supervised learning model is trained to map images to a fixed set of class labels. Hence, supervised learning models cannot accurately predict classes it was not trained on.

In contrast, using ZSL a model can classify images belonging to previously unseen classes with a reasonable accuracy. This is because a self-supervised foundation model like CLIP is pretrained using a very large image and corresponding caption corpus that contains a vast array of contexts (such as “A group of people drinking beer at a bar.”, and “Celebrating end of exams party with friends at a pub and drinking cocktails.”). Since CLIP learned to associate each image to the contextual features of a phrase (like “bar”, “drinking”, “people” and “beer”) during pretraining, implementing ZSL on CLIP does not require any additional training for classifying an image into a new class. Therefore, we need to select relevant phrases for each label to achieve high ZSL performance. Results presented in this paper (see “Results” section) show extensive phrase engineering can measurably improve ZSL performance.

To do phrase engineering for ZSL, a small, labelled validation set is required. In contrast, supervised learning additionally requires a large, labelled training dataset which takes extensive manual effort to assemble and annotate. Furthermore, supervised learning usually requires a machine learning developer to write code which organises the data and trains a model. In addition, supervised learning can be computationally expensive particularly when training deep complex models on large datasets.

### Procedure and dataset

In this paper, we compared the ZSL performance of CLIP against supervised learning (using ABIDLA2) on the test dataset introduced in the ABIDLA2^12^ paper. The CLIP model we used is a transformer network model with 151.28 million parameters that requires maximum memory of 344.85MiB for processing a batch of 1 image, whereas ABIDLA2 is a convolutional neural network model with 6.963081 million parameters that requires maximum memory of 207.83 MiB for processing a batch of 1 image. The dataset consisted of eight beverage categories consisting of seven alcoholic beverage categories and the "others” category.

Upon closer inspection of the original ABIDLA2 dataset (which we will call ABD-2022), we found a substantial proportion of the images in the “others” category of the test set contained alcoholic beverage categories such as gin or vodka that were not included in ABIDLA2. Hence, to delineate images more clearly without alcohol-related content, we relabelled the “others” category in the test dataset manually using two different annotators and only kept the images that both annotators agreed belongs in the non-alcohol related “others” category. In this modified dataset (called ABD-2023), we removed 1,177 alcohol-related images from the “others” category and replaced them with 1,177 Google images using the following search terms: “sports cars”, “architecture”, “seascape”, “villas”. These added images were manually checked to ensure that they belonged in the non-alcohol related “others” category. The images in the remaining test dataset categories remained unchanged from ABD-2022, as did all the training and validation examples.

Table 1 shows the number of images in the training, validation, and testing datasets for the ABD-2023 dataset that we used for the comparison between ABIDLA2 and ZSL. To maintain a uniform testing set distribution there were exactly 1,762 images per class.

**Table 1:** Number of images in the training, validation, and testing splits of the ABD-2023 dataset.

Beverage class	Training dataset	Validation dataset	Testing dataset
Beer/Cider cup	11,485	1016	1762
Beer/Cider bottle	16,591	1138	1762
Beer/Cider can	7721	535	1762
Wine	16,583	1279	1762
Champagne	10,207	606	1762
Cocktails	14,736	1006	1762
Whiskey/Cognac/Brandy	37,543	2117	1762
Other images	49,805	4822	1762
Total	164,671	12,519	14,096

To maintain uniform distribution in the testing dataset, we kept 1762 images per beverage class.

### Zero-shot learning model

**Figure 1:**
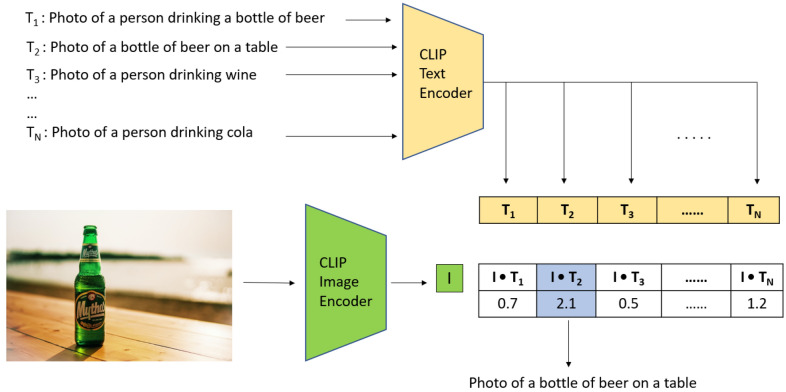
Example showing how the CLIP text encoder and image encoders are used to perform zero-shot classification on our dataset of alcoholic beverage images. Here, “I” is the vector representation of the image, and T_1_…T_N_ are vector representations of predetermined text phrases.

We used the pre-trained CLIP^13^ model to implement ZSL on the test dataset using methods prescribed in the CLIP paper 13. Figure 1 shows how we used the image encoder and text encoder of the CLIP model^13^ to perform zero-shot classification. First, we represent each class using a single phrase or a group of phrases. For example, the phrases used for the beer bottle class can be a single phrase (such as “beer bottle”) or a group of phrases that describes a context in which the beverage is portrayed (such as “photo of a person drinking a bottle of beer” and “photo of a bottle of beer on a table”). Then we feed each phrase into the text encoder of the CLIP model^13^ to generate a vector representation for each phrase, which is a condensed sequence of numbers that represents the semantic content of the phrase. Next, an input image is fed into the image encoder of the CLIP^13^ model to generate a vector representation for the image, which is comparable with the vector representations of phrases. The vector representing the image is then multiplied by each phrase vector to arrive at a similarity measure between the image and each phrase. We then select the class associated with the phrase with the highest similarity measure as the predicted class.

### Phrase engineering for zero-shot learning

Recent artificial intelligence (A.I.) models such as ChatGPT^14^ and Stable Diffusion^15^ that have attracted a widespread userbase have implemented a technique called “prompt engineering”. Prompt engineering is the deliberate act of users wording input prompts in a specific way such that the A.I. model produces more desirable results. For example, users have found that including terms such as “4 k resolution” and “award-winning photography” in their input prompts led to generate higher quality images. Similarly, the ZSL performance of models is sensitive to the specific phrases used to represent each class and we refer to the act of carefully selecting such phrases for ZSL as “phrase engineering”. For example, using the term “beer bottle” instead of a phrase “photo of a person drinking a bottle of beer” may lead to worse results in identifying images of a beer bottle in a social context since the ZSL models (like CLIP) are usually pretrained on descriptive captions of images rather than one- or two-word phrases (in this case contextless class names). Hence it is important to find appropriate *descriptive phrases* that represent each class. We have therefore used our labelled validation set of 12,519 images for finding the locally optimal set of *descriptive phrases* for each class. This is done by evaluating model performance using various *descriptive phrases* until the locally optimal set of *descriptive phrases* that yield best performance for each class were found. Note that only the validation dataset was used for phrase engineering, and the test dataset was completely hidden from the ZSL model until the final evaluation. It should also be noted that finding a globally optimal set of descriptive phrases that covers all contexts is virtually impossible due to the myriad of possible descriptions per each class, hence we propose that users take a heuristic approach and try different phrases for each class, then test their effectiveness using a labelled validation dataset.

To investigate the sensitivity of ZSL to the phrases used to represent each beverage category, we tested two different approaches. The first approach just uses the beverage names and their containers exactly as they were referred to in ABIDLA2^12^ as class labels, such as “Beer/Cider Cup”, “Wine”, and “Whiskey/Cognac/Brandy”. We call these the *name-based phrases*.

In the second approach, multiple *descriptive phrases* were used to represent each beverage category. For example, the “beer/cider bottle” class was represented by the following *descriptive phrases*: “photo of a person drinking a bottle of beer” and “photo of a bottle of beer on a table”. So, if either of these two phrases match the image then the image is predicted to be in “beer/cider bottle” class. Using multiple phrases to describe the same class should give better results since alcoholic beverages can appear in different settings, e.g., sometimes a person is actively drinking from a beer bottle and other times a beer bottle is just sitting on a table. Having phrases that better match the setting will likely mean the image will be more strongly associated with the phrase and less likely to match an unrelated phrase instead. However, it is important to note that it is not necessary (and a virtually impossible task) to enumerate all possible settings that an alcoholic beverage can appear in, since in general the type of beverage (e.g., beer versus wine) should still be a predominant factor in determining where the phrase vector is positioned in the vector space. For similar reasons we did not find it necessary to enumerate all types of alcoholic beverages within a category (i.e., cider in addition to beer; cognac and brandy in addition to whiskey). Due to the visual similarities among the types of alcoholic beverages (such as beer and cider) within a category the additional phrases were not found to increase performance. For example, the phrase “photo of a person drinking a bottle of beer” matches sufficiently to images of people drinking cider.

While performing phrase engineering, we found it particularly challenging to create the *descriptive phrases* to capture the entirety of the “others” class, since the “others” class effectively represents any image that has no alcoholic beverages. For example, if we just use the phrase “others” to represent the “others” class and are given an image of someone drinking from a coke bottle, it may be the case that the image will be associated with the phrase “A person drinking from a beer bottle” because most of the content of the image will match the bottle drinking part of the phrase. Whereas the “others” phrase which is more generic may be mapped to somewhere further away in the vector space. A way of thinking about this is that in this case the “others” phrase is just a single point in a huge vector space, so it is hard to ensure all non-alcoholic images are closest to this single point rather than the set of points representing all the other classes. It is for this reason that we opted to create a very extensive list of phrases for the “others” class when using *descriptive phrases*. Table 2 shows the set of *name-based phrases* and *descriptive phrases* used to represent each class.

**Table 2:** Table showing the set of name-based phrases and there corresponding descriptive classes.

ABDILA 2 class	Name-based phrase	Descriptive phrase
Beer/cider cup	Beer_or_cider_cup	Photo of a person drinking a glass of beer; photo of a glass of beer on a table
Beer/cider bottle	Beer_or_cider_bottle	Photo of a person drinking a bottle of beer; photo of a bottle of beer on a table
Beer/cider can	Beer_or_cider_can	Photo of a person drinking a can of beer; photo of a can of beer on a table
Wine	Wine	Photo of a person drinking wine; photo of wine on a table
Champagne	Champagne	Photo of a person drinking champagne; photo of champagne on a table
Cocktails	Cocktails	Photo of a person drinking a cocktail; photo of a cocktail on a table
Whiskey/cognac/brandy	Whiskey_cognac_or_brandy	Photo of a person drinking whiskey; photo of a person drinking a glass of whiskey; photo of whiskey on a table
Others	Other	Photo of a person drinking from a plastic bottle; photo of a person drinking * (10 phrases with * replaced with cola, soda, a soda can, juice, water, a glass of water, a smoothie, coffee, tea, and milk; photo of * (18 phrases with * replaced with the ground, a single can of coca-cola, fruit, an animal, a room, text, writing, food, a person, a logo, furniture, a glass, clothing, a drawing, a jar, a building, a straw, and an object)

### Data analysis

Using our test dataset, we created three separate tasks for evaluating the performance of the ZSL vs ABIDLA2. Task 1 is to classify any given image into one of the eight specific categories: Beer/Cider Cup, Beer/Cider Bottle, Beer/Cider Can, Wine, Champagne, Cocktails, Whiskey/Cognac/Brandy, Others. Task 2 is to classify any given image into one of four broader categories: Beer (Beer/Cider cup, Beer/Cider Bottle, Beer/Cider Can classes merged); Wine (Wine and Champagne classes merged); Spirits (Cocktails and Whiskey/Cognac/Brandy classes merged); and Others. Task 3 is a binary classification problem with the following two classes: Alcoholic Beverages, and Others. We compared the performance metrics of ABIDLA2, ZSL using *name-based phrases*, ZSL using *descriptive phrases* across the three tasks. In addition, we also computed three separate confusion matrices to analyse the performance of each of ABIDLA2, ZSL using *name-based phrases*, ZSL using *descriptive phrases* vs annotators labels.

We report results for the following three metrics: unweighted average recall (UAR), F1 score and per class recall. We report the UAR metric instead of accuracy since for Task 2 and 3 the class distributions are skewed as a result of merging; hence accuracy would be dominated by how well the model predicts the majority class (Beer for Task 2 and Alcoholic Beverage for Task 3).

## Results

### Task 1: Classification of eight specific categories

**Figure 2:**
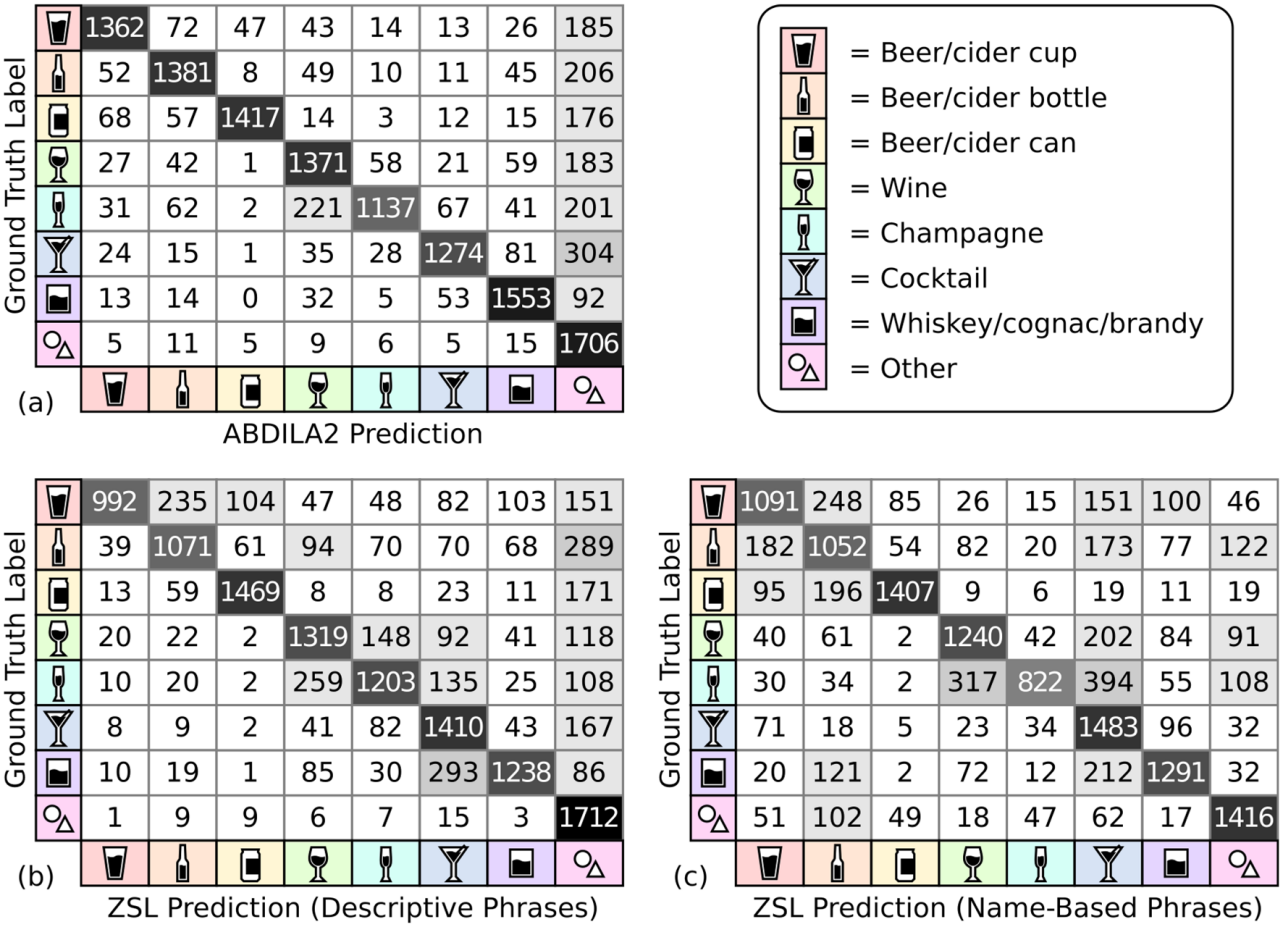
Confusion matrices comparing model predictions with ground truth labels (manual annotations) for Task 1. The numbers represent the number of test set examples with that combination of ground truth label and model prediction; hence the main diagonal represents correct predictions.

**Figure 3:**
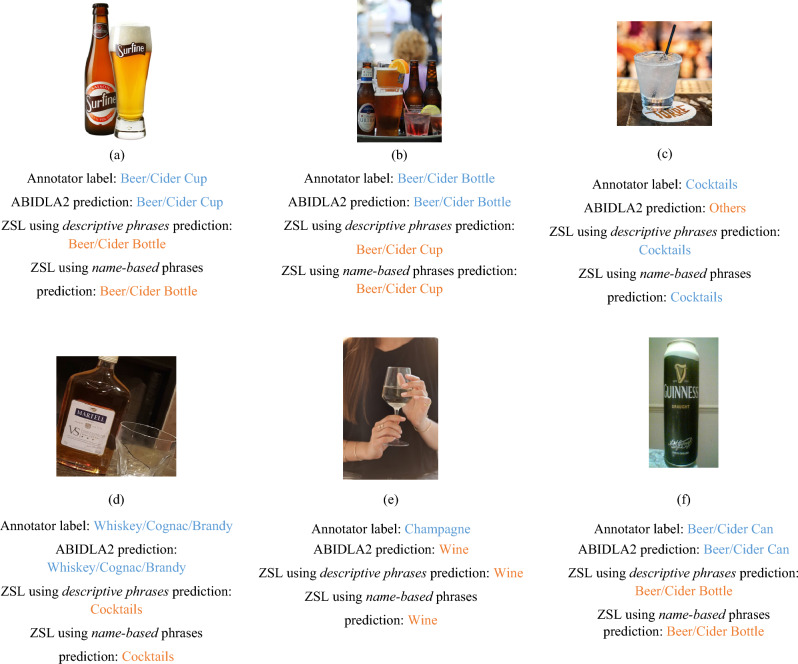
Visualizations of the predictions made by ABIDLA2, ZSL using *name-based* phrases, ZSL using *descriptive phrases* in comparison to annotator labels. The above images are posted for educational purposes only.

As seen in Table 3, overall ABIDLA2 clearly outperformed both ZSL using *name-based phrases* and ZSL using *descriptive phrases* on Task 1. In order to analyse the cause of the performance disparity in more detail, the confusion matrices for the three approaches (Fig. 2) show that a main contributor to ABIDLA2 achieving a greater UAR and F1 score in Task 1 is that it is better than ZSL models at (1) separating beer cans from beer bottles and beer cups (see Fig. 3a,b); and (2) separating the different types of spirits. The most frequent mistake ABIDLA2 made was classifying a cocktail as "Others" (see Fig. 3c), whereas the most frequent mistake made by ZSL using *descriptive phrases* model was classifying whiskey/cognac/brandy as "Cocktails" (see Fig. 3d). Both ZSL and ABIDLA2 struggled with champagne being misclassified as "Wine" (see Fig. 3e). The *descriptive phrases* generally result in better predictions from the ZSL model than the *name-based phrases*, particularly with respect to recall for the "Others" category. That is, the *name-based phrases* are much more likely to result in a non-alcohol-related image to be erroneously predicted as having alcohol content (with "beer/cider bottle" being the most frequent).

**Table 3:** Comparison between ABIDLA2, ZSL using descriptive phrases and ZSL using name-based phrases.

	ABIDLA2	ZSL using descriptive phrases	ZSL using name-based phrases	ABIDLA2	ZSL using descriptive phrases	ZSL using name-based phrases
	Recall	Recall	Recall	F1 score	F1 score	F1 score
Task 1						
Beer/Cider cup	77.3	56.3	61.92	81.46	69.49	65.29
Beer/Cider bottle	78.38	60.78	59.7	80.85	66.81	58.54
Beer/Cider can	80.42	83.37	79.85	87.39	86.11	83.55
Wine	77.81	74.86	70.37	77.55	72.85	69.88
Champagne	64.53	68.27	46.65	75.22	71.65	59.57
Cocktails	72.3	80.02	84.17	79.18	72.64	66.53
Whiskey/Cognac/Brandy	88.14	70.26	73.27	86.35	75.17	73.92
Others	96.82	97.16	80.36	70.86	75.02	78.06
**UAR/Macro** **F1** **score**	**79.46**	73.88	69.54	**79.86**	73.72	69.42
Task 2						
Beer	84.45	76.49	83.43	89.25	85.36	85.6
Wine	79.09	83.12	68.7	84.98	83.94	76.75
Spirits	84.02	84.68	87.46	86.9	83.17	77.52
Others	96.82	97.16	80.36	70.86	75.02	78.06
**UAR/Macro** **F1** **score**	**86.1**	85.36	79.99	**83**	81.87	79.48
Task 3						
Non-alcoholic beverages	96.82	97.16	80.36	70.86	75.02	78.06
Alcoholic beverages	89.08	91.16	96.35	94	95.18	96.76
**UAR/Macro** **F1** **score**	92.95	**94.16**	88.36	82.43	85.1	**87.41**

The values in the table represent recall values of each class to a total of 100%. Bold values indicate the best model results for each task.

### Task 2: Classification of four broad categories

For Task 2, ABIDLA2 only marginally outperformed the ZSL using *descriptive phrases* model, whereas ABIDLA2 clearly outperformed the ZSL using *name-based phrases* model (see Table 3). A common cause of confusion for the ZSL model in Task 1 was between visually similar depictions of alcohol (e.g., beer/cider in different vessels as shown in Fig. 3f and wine/champagne as shown Fig. 3e), as evidenced by the confusion matrices (Fig. 2). Since these similar classes were merged for Task 2, the gap in UAR and F1 score between ZSL using descriptive phrases and ABIDLA2 reduced substantially. A key contributor to the slightly better performance of ABIDLA2 was the tendency for ZSL using *descriptive phrases* to incorrectly classify images depicting wine or champagne as "Cocktails".

### Task 3: Binary classification

In Task 3 (classifying images as alcohol-containing or not), the ZSL using *descriptive phrases* outperformed both ABIDLA2 and ZSL using *name-based phrases* by 1.21% and 5.8% UAR, respectively (see Table 3). Similarly, the ZSL using *name-based phrases* outperformed both ABIDLA2 and ZSL using *descriptive phrases* by 4.98% and 2.31% F1 score, respectively (see Table 3). In this binary classification task, although ZSL outperformed ABIDLA2, there are different trade-offs for name-based and descriptive phrases. Namely, descriptive phrases algorithm has more true negatives, but this is at the expense of a larger number of false negatives. Table 4 clearly presents information about this trade-off between ZSL models.

**Table 4:** Task 3 results presented in terms of binary classification metrics, with “alcoholic beverages” as the positive case. “↑” indicates higher is better, “↓” indicates lower is better.

	TP ↑	FN ↓	TN ↑	FP ↓	Precision ↑	recall ↑	UAR ↑	F1-score ↑
ABIDLA2	10,987	1347	1706	56	0.9949	0.8908	0.9295	0.9400
ZSL using descriptive phrases‍	11,244	1090	**1712**	**50**	**0.9956**	0.9116	**0.9416**	0.9518
ZSL using name-based phrases	**11,884**	**450**	1416	346	0.9717	**0.9635**	0.8836	**0.9676**

Bold values indicate the best model results. TP represents True Positives, FN represents False Negatives, TN represents True Negatives and FP represents False Positives.

## Discussion

In this article, we investigated the performance of zero-shot learning (ZSL) compared to a deep learning algorithm specifically trained to identify alcoholic beverages in images (ABIDLA2)^12^. In addition, we also investigated whether ZSL using *descriptive phrases* performed better than ZSL using *name-based phrases*. The results show that ZSL performed worse, similar, and better than ABIDLA2 in identifying alcoholic beverages from digital images in Task 1, 2, and 3, respectively. Specifically, we found that the ZSL using *descriptive phrases* (e.g., “this is a picture of someone holding a beer bottle”) performed nearly as well as ABIDLA2 on classifying specific beverages into broader beverage categories (beer, wine, spirits, and other, i.e., Task 2) and even surpassed ABIDLA2 when classifying whether a picture included alcohol content or not (Task 3). However, ABIDLA2 outperformed ZSL using *descriptive phrases* in fine-grained classification task i.e., recognising specific alcoholic beverages and containers (e.g., beer/cider bottles, cups) (Task 1). In task 1 and 2, in terms of UAR and F1 score, ZSL using *named-based phrases* performed worse than ZSL using *descriptive phrases* and ABIDLA2. However, in task 3 (in terms of F1 score), ZSL using *named-based phrases* performed better than ZSL using *descriptive phrases* and ABIDLA2.

It may seem surprising that ZSL, without any specific training on alcohol-containing images, can outperform ABIDLA2 in any of the classification tasks, since ABIDLA2 is a bespoke model trained explicitly for alcohol image recognition. However, the ZSL model (CLIP) has been pretrained on a general dataset of 400 million captioned images in contrast to the 164,671 images that ABIDLA2 was trained on. Although the ZSL training was not alcohol-specific, it does mean that the ZSL model has a more complete internal representation of image composition in general and can therefore more easily generalise to images which have not yet been seen by the model. In contrast, ABIDLA2 performed better at identifying more subtle differences among different types of alcoholic beverages and containers (Task 1) as it was trained in a more focused way (see Fig. 3a,b,f).

ZSL using *descriptive phrases* outperformed ZSL using *name-based phrases* in all three tasks in terms of UAR. This indicates the importance of phrase engineering for ZSL models for achieving higher performance in terms of UAR. The importance of phrase engineering is particularly prominent for the "Others” class since the ZSL using *name-based phrases* performed considerably worse than ZSL using *descriptive phrases* model for the “Others” class for all three tasks. This is because it is hard to find a single point in the vector space to represent all other classes since, for example, a coke bottle may be closer to beer bottle in the vector space than to the “Others” phrase. The ZSL using *descriptive phrases* used 38 phrases to represent the “Others” class.

Although we identified that phrase engineering is essential for ZSL in terms of achieving higher performance, it must be noted that the ZSL models are inclined to be confused over fine-grained classification among beverage categories with higher similarity. For example, as our results indicate, the ZSL models are confused between “beer bottle” and “beer cup” (see Fig. 3a); and between “wine” and “champagne” (see Fig. 3e). This could be because the text encoder used in the CLIP model is likely to embed similar things (like wine and champagne) closer to each other than unrelated things (like wine and robots) in the overall vector space. Consequently, if the aim is discriminating between very similar concepts which differ only in subtle aspects like the kind of container used for the same type of beverage, it may be better to create a labelled dataset to train a supervised model specifically for this task rather than using ZSL.

### Limitations, strengths and recommendations for future research

We only used the pretrained CLIP model^13^ to implement ZSL as it was available at the time of this study. In future works, it would be interesting to try out more recent pretrained foundation models trained on different data (such as Google’s CoCa^16^) for ZSL which may offer better accuracy or speed.

The fact that the ZSL model that was not exclusively trained on ABD-2023 dataset but still performed similarly or even better than ABDILA2, a bespoke model that is trained on ABD-2022, suggests that ZSL will likely generalise better than ABDILA2 to other datasets from different sources (such as Instagram, Facebook, and Twitter) containing images of alcoholic beverages. Hence an important direction for future work is to compare the generalisation capability of supervised learning models to ZSL on real-life datasets that include images of different populations and cultures. Since foundation models like CLIP are trained on publicly available images on the internet, they may be biased towards certain images of alcoholic beverages that are most popular (e.g., what is prominent in Western culture). As such, the models may be less accurate at identifying alcohol-related images, particularly when non-Western forms of alcoholic beverages or containers are portrayed.

### Implications for alcohol research and policy

This study demonstrates that ZSL—an approach that requires virtually no additional training data, less computational resources, and less computer science expertise than supervised deep learning—can accurately address research questions such as the identification of alcohol content in images, especially when binary classification is required (i.e., identifying the presence/absence of alcoholic beverages). To date, most estimates of alcohol exposure in media are based on self-report—which is subject to recall bias—or hand-annotated data—which is time-intensive and difficult to conduct at large scale^10^. As such, ZSL could assist with determining more accurate estimates of alcohol exposure in the media, which is important for determining how common alcohol is across media platforms, how alcohol is being portrayed, and what impact alcohol exposure may have.

## Conclusion

This paper has shown that ZSL—without being explicitly trained using labelled images of alcoholic beverages—can achieve similar or even slightly better results compared to the supervised learning used by ABIDLA2 for classifying between alcoholic beverages and other images. This is a remarkable achievement since ABIDLA2 was explicitly trained for this purpose on 164,671 labelled images. The fact that ZSL performed so well even though the model was not trained on the specific labels used for downstream alcoholic beverage recognition tasks suggests that ZSL models are likely to generalise better to a new dataset than ABIDLA2 would. However, it is important to note that deploying a ZSL model requires some effort in phrase engineering. Since our results indicate that using contextless *name-based phrases* (e.g., ‘wine’) to represent each class results in considerably poorer performance (in terms of UAR) compared to using multiple *descriptive phrases* (which contains contextual features) per class. To find effective phrases, a small, labelled validation set is required to test different phrase candidates against each other.

The fact that implementing ZSL on new datasets require little to no prior programming experience can revolutionise the way public health researchers can analyse vast amounts of data efficiently to obtain alcohol exposure analytics from digital images. Such analytics can further be used to provide insights (like demonstrating the amount of brand-specific alcohol exposure in social media sites like Facebook, Instagram, and Twitter) based on which researchers and policy makers can propose regulations to prevent alcohol exposure and eventually alcohol consumption and related harm.

## Data Availability

The datasets used in the current study are available from the corresponding author on reasonable request.
